# Multi-scale systems toxicology defines a KLF5-centered adverse outcome pathway linking DEHP exposure to pancreatic cancer progression and signaling programs relevant to therapy tolerance

**DOI:** 10.3389/fcell.2026.1769023

**Published:** 2026-02-05

**Authors:** Jihao Chen, Huaxin Pang, Jundan Wang, Junhua Guo, Ting Huang, Heran Zhou

**Affiliations:** 1 Department of Oncology, Hangzhou TCM Hospital Affiliated to Zhejiang Chinese Medical University, Hangzhou, Zhejiang, China; 2 Chinese Medicine Data Center, China Academy of Chinese Medical Sciences, Beijing, China

**Keywords:** di (2-ethylhexyl) phthalate, KLF5, pancreatic ductal adenocarcinoma, phthalates, PI3K–AKT signaling, systems toxicology, TGF-β signaling, tumor immune microenvironment

## Abstract

Di(2-ethylhexyl) phthalate (DEHP) is a ubiquitous plasticizer implicated in pancreatic carcinogenesis, yet the molecular initiating events and adverse outcome pathways (AOPs) linking exposure to disease mechanisms remain poorly resolved. In this study, we integrated a multi-scale systems toxicology framework—combining heterogeneous ensemble machine learning, Mendelian randomization (MR), molecular docking and molecular dynamics (MD), single-cell transcriptomics, and *in vitro* assays—to delineate a candidate mechanistic trajectory. A Tabular Prior-Data Fitted Network (TabPFN)-enhanced ensemble identified a six-gene pancreatic ductal adenocarcinoma (PDAC) signature (AUC = 0.946). Within this signature, MR provided suggestive evidence for a modest association between genetically predicted Krüppel-like factor 5 (KLF5) expression and pancreatic cancer risk (OR = 1.188, *p* = 0.046). Functional enrichment of DEHP–PDAC intersection targets highlighted pro-survival signaling modules, including PI3K–AKT- and MAPK-related pathways. Structure-based analyses supported the biophysical plausibility of a non-covalent DEHP–KLF5 interaction (−6.4 kcal/mol), and 100-ns MD simulations indicated a persistent binding mode with conformational accommodation. Single-cell analysis localized KLF5 predominantly to malignant ductal cells and, together with CellChat inference, was consistent with malignant cell-derived TGF-β- and MIF-related signaling. Network-based virtual knockout further suggested that KLF5 may contribute to sustaining a TGF-β/MMP7-linked matrix remodeling program. Consistently, DEHP exposure upregulated KLF5 and MMP7 and enhanced migration and invasion of PANC-1 cells. Collectively, these findings support a working AOP model linking DEHP-responsive KLF5-centered activity to extracellular matrix (ECM) remodeling and immunomodulatory communication, providing a mechanistic rationale that aligns with signaling programs characteristic of therapy-tolerant tumor niches, particularly PI3K–AKT-coupled survival signaling and TGF-β-linked stromal remodeling. Although these implications are hypothesis-generating, they highlight a potential avenue for future drug-response investigations.

## Graphical Abstract

**Figure:**
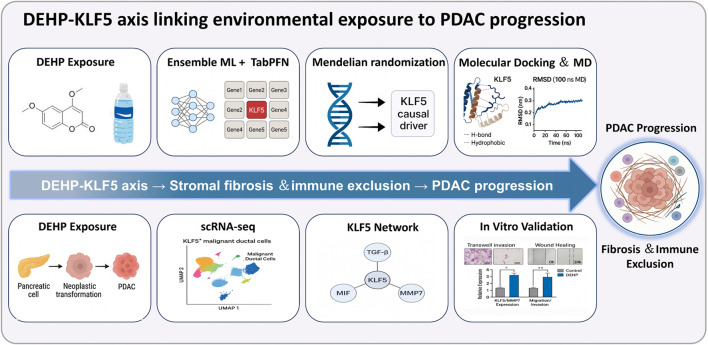

## 1 Introduction

Pancreatic ductal adenocarcinoma (PDAC) remains one of the most lethal malignancies worldwide, with an insidious clinical course and a 5-year survival rate below 10% (Sung et al., 2021). Although inherited susceptibility contributes to a risk, the concurrent increase in PDAC incidence alongside industrialization has renewed interest in the “exposome”—the cumulative burden of environmental chemical exposures—as a modifiable contributor to pancreatic tumorigenesis (Wild, 2012). Di(2-ethylhexyl) phthalate (DEHP), a high-production plasticizer widely used in polyvinyl chloride (PVC) products, is of particular concern because it can leach into food, water, and medical fluids, enabling sustained human exposure.

DEHP is rapidly metabolized to mono(2-ethylhexyl) phthalate (MEHP), its primary bioactive metabolite. Despite the long-standing recognition of DEHP/MEHP as endocrine-disrupting chemicals (EDCs), their effects on the exocrine pancreas remain less well characterized than those on reproductive and metabolic systems (Martínez-Pinna et al., 2023). Epidemiological studies have linked phthalate burden to PDAC-related precursor conditions, including chronic pancreatitis and type 2 diabetes (Mariana and Cairrao, 2023), and experimental work suggests that phthalates can elicit oxidative and inflammatory signaling in pancreatic tissue (Lin et al., 2011; Baralić et al., 2020). Mechanistic evidence, however, has often centered on β-cell dysfunction (Sun et al., 2015). In contrast, direct evidence in pancreatic ductal/epithelial models remains relatively scarce, and the ductal-specific stress signaling and differentiation programs engaged by DEHP/MEHP are not well delineated. This gap motivates a focused assessment of ductal-relevant molecular initiating events and their downstream microenvironmental consequences. Because PDAC arises predominantly from the ductal lineage (or via acinar-to-ductal metaplasia), a key unresolved question is how DEHP-related stress perturbs ductal homeostasis in ways that facilitate malignant transformation. Although MEHP is often treated as the principal effector, in this study, DEHP is used as the index environmental stressor to anchor adverse outcome pathway (AOP) construction; metabolite-inclusive models (such as MEHP and mixed phthalate exposures) remain important for subsequent refinement.

AOP development requires a molecular initiating event (MIE), which is defined as the first measurable interaction between a chemical stressor and a biological target that propagates downstream key events (KEs) (Ankley et al., 2010). For DEHP-associated PDAC, this initiating bridge has not been clearly established. Krüppel-like factor 5 (KLF5) is a plausible candidate: it is a core transcriptional regulator of epithelial/ductal programs and PDAC progression, with reported associations with lineage plasticity and aggressive phenotypes (Zhou Y. et al., 2025). As a transcription factor, KLF5 is dynamically regulated by upstream kinase signaling and post-translational modifications (phosphorylation and ubiquitin-dependent turnover), which can alter its stability, subcellular localization, and cofactor interactions. Accordingly, KLF5 could plausibly be perturbed by chemical stress indirectly through these upstream regulatory layers or, as a testable hypothesis, via a direct interaction that is amenable to structure-based evaluation. We, therefore, asked whether DEHP exposure could engage a KLF5-centered regulatory axis—indirectly through upstream signaling that modulates KLF5 stability and function or through a direct chemical–protein interaction that can be evaluated structurally—thereby shaping downstream programs relevant to extracellular matrix (ECM) remodeling and immune contexture within the tumor immune microenvironment (TIME).

Addressing this question benefits from an integrative design that spans target prioritization, genetic support, structural plausibility, cellular localization, and functional readouts. Accordingly, we combined a Tabular Prior-Data Fitted Network (TabPFN)-enhanced heterogeneous ensemble learning strategy to prioritize candidate targets (Hollmann et al., 2025), Mendelian randomization (MR) to assess genetic support for target–disease relationships (Gill et al., 2019), molecular docking and molecular dynamics (MD) to evaluate the biophysical plausibility of candidate chemical–protein interactions, and single-cell analyses together with *in vitro* assays to localize target activity and test phenotypic consequences. Because stromal remodeling and immune exclusion are frequently implicated in treatment-refractory PDAC, defining exposure-responsive signaling programs that plausibly reinforce such niches may inform therapy-relevant hypotheses, even when drug response is not directly assayed.

In this study, we constructed a mechanism-informed AOP linking DEHP exposure to PDAC progression by integrating chemical-target mining with PDAC transcriptomic signals, cross-validated heterogeneous ensemble learning, genetic causal inference, and structure-based modeling of a candidate MIE. We then localized KLF5 activity using single-cell RNA sequencing (scRNA-seq), derived testable downstream programs via network-based perturbation analysis, and performed *in vitro* validation of tumor-intrinsic key events (KLF5/MMP7 induction and pro-migratory/pro-invasive phenotypes) in DEHP-exposed PANC-1 cells. Together, these analyses provide a hypothesis-driven basis for AOP refinement and support hazard identification for DEHP and related phthalates.

## 2 Materials and methods

### 2.1 Data acquisition and target identification

The chemical structure and canonical Simplified Molecular Input Line Entry System (SMILES) of DEHP were retrieved from PubChem (https://pubchem.ncbi.nlm.nih.gov/). Potential DEHP-interacting targets were identified by screening ChEMBL (https://www.ebi.ac.uk/chembl/), STITCH (http://stitch.embl.de/), and SwissTargetPrediction (http://swisstargetprediction.ch/), with the organism restricted to *Homo sapiens*. Pancreatic cancer-associated targets were concurrently collated from the Online Mendelian Inheritance in Man (OMIM) (https://omim.org/) and GeneCards databases (https://www.genecards.org/). Intersection analysis was performed to identify candidate genes potentially bridging DEHP exposure and pancreatic tumorigenesis.

### 2.2 Functional enrichment analysis

Gene Ontology (GO) and Kyoto Encyclopedia of Genes and Genomes (KEGG) pathway enrichment analyses were conducted using the DAVID database. Biological processes (BPs), cellular components (CCs), and molecular functions (MFs) were analyzed. Statistical significance was defined as a Benjamini–Hochberg-adjusted *p*-value <0.05.

### 2.3 Construction of a heterogeneous ensemble learning framework

To overcome the selection bias inherent in single-model approaches and capture diverse data structures, we developed a heterogeneous ensemble machine learning (ML) framework using transcriptomic data from the GSE15471 dataset. This framework integrated eight distinct algorithms, spanning classical linear classifiers [logistic regression and support vector machine (SVM)] and robust tree-based methods (random forest, GBDT, and XGBoost). Notably, we incorporated a novel transformer-based algorithm, TabPFN. Unlike traditional ML models, TabPFN leverages in-context learning pre-trained on synthetic datasets, offering superior robustness, particularly for small-to-medium-sized biological datasets. Model performance was rigorously evaluated using 5-fold cross-validation.

### 2.4 SHAP interpretability analysis

To address the “black-box” weakness of complex machine learning models, we used SHapley Additive exPlanations (SHAP) to interpret the diagnostic framework (Qi et al., 2025). SHAP values were calculated for each of the eight algorithms to quantify the marginal contribution of individual core genes to the model’s prediction. A positive SHAP value indicates a positive correlation with disease probability, while a negative value suggests a protective effect. We aggregated these values to visualize the global importance of the identified core targets, thereby validating their biological relevance and diagnostic potential.

Furthermore, to distill the most robust molecular signature, we implemented a stringent frequency-based intersection strategy. Feature (i.e., gene) importance rankings were independently derived from each of the eight algorithms. We chose the top five-ranked features for each algorithm. The final core biomarkers were defined as those consensus genes identified as top-prioritized features by at least seven of the eight algorithms (frequency ≥7/8). By demanding consistency across highly heterogeneous architectures—from linear planes to decision trees and transformers—this strategy effectively filters out algorithm-specific artifacts, ensuring the identification of biologically stable and generalizable targets (Wang et al., 2022).

### 2.5 External validation in TCGA and GTEx cohorts

External validation was conducted using the UCSC Xena uniformly processed Toil RNA-seq (TCGA–TARGET–GTEx) dataset to reduce technical variability between TCGA–PAAD tumor samples (n = 179) and GTEx normal pancreas tissues (n = 171). Expression values were transformed as log_2_(TPM + 1). Between-group differential expression was assessed using a two-sided Wilcoxon rank-sum test. For survival analyses, TCGA–PAAD patients with available overall survival data (n = 178) were dichotomized into high- and low-expression groups using the median expression of each gene. Overall survival was evaluated using Kaplan–Meier curves with the log-rank test, and hazard ratios (HRs) were estimated using univariate Cox proportional hazards models.

### 2.6 Immune microenvironment profiling

The tumor immune landscape was characterized by quantifying the relative proportions of 22 infiltrating immune cell types using the CIBERSORT algorithm (Ge et al., 2019) with 1,000 Monte Carlo permutations. Samples with a deconvolution *p*-value <0.05 were retained. Spearman’s rank correlation analysis was performed to evaluate regulatory relationships between core gene expression and immune cell abundance.

### 2.7 Two-sample Mendelian randomization analysis

We used two-sample MR to infer causal relationships between core gene expression (exposure) and pancreatic cancer risks (outcome). Genetic instruments (cis-eQTLs) were derived from the eQTLGen Consortium (n = 31,684), and pancreatic cancer summary statistics were obtained from the FinnGen Consortium (R12 release). Instrumental variables (IVs) were selected based on genome-wide significance (*p* < 5 × 10^−8^). To ensure independence, SNPs were clumped using a linkage disequilibrium (LD) threshold of r^2^ < 0.01 within a 10,000 kb window, based on the 1000 Genomes Project European reference panel. Instrument strength was confirmed via F-statistics (>10). The inverse-variance weighted (IVW) method was used as the primary analysis, with MR–Egger and weighted median analyses performed as sensitivity analyses to evaluate the robustness of causal estimates under different instrument-validity assumptions. Horizontal pleiotropy was assessed using the MR–Egger intercept test and MR–PRESSO, and heterogeneity was evaluated using Cochran’s Q statistic (Boehm and Zhou, 2022).

### 2.8 Molecular docking and molecular dynamics simulations

Core target protein structures were retrieved from the Protein Data Bank (PDB; http://www.rcsb.org/). For KLF5, the experimentally determined human KLF5 zinc-finger DNA-binding domain structure (PDB ID: 2EBT) was used for docking and MD simulations as it represents the functionally essential DNA-binding region and is currently the highest-confidence human KLF5 structure available. The DEHP structure was obtained from PubChem. Molecular docking was performed using AutoDock Vina, and conformations with the lowest binding energies were visualized using PyMOL 3.2 (Eberhardt et al., 2021).

To assess the *in silico* stability of the DEHP–KLF5 complex, a 100-ns MD simulation (Lemkul, 2024) was performed using GROMACS 2020.6. Given the current lack of a full-length KLF5 structure, the zinc-finger DNA-binding domain (PDB ID: 2EBT) was selected as the best-available template for domain-level modeling. The complex was parameterized with the CHARMM36m force field (protein/Zn^2+^) and CGenFF (ligand) in a TIP3P water box. Following energy minimization, the system underwent NVT and NPT equilibration. The 100-ns production run was maintained at a constant temperature of 300 K (Nosé–Hoover thermostat) and a pressure of 1 bar (Parrinello–Rahman barostat). Long-range electrostatics was treated via PME, and covalent bonds were constrained using the LINCS algorithm. Trajectory analyses, including RMSD, RMSF, Rg, and hydrogen bonding, were conducted to characterize conformational dynamics. These computational analyses provide structural plausibility evidence under domain-specific constraints rather than experimental confirmation of binding. Principal component analysis (PCA) was performed to extract dominant motions, and the free energy landscape (FEL) was constructed based on the first two principal components (PC1 and PC2).

### 2.9 Single-cell RNA sequencing and interaction analysis

The scRNA-seq dataset GSE291124 (four PDAC tumor samples) was processed using Seurat (v5.0) (Hao et al., 2021). Quality control excluded cells with gene counts <200 or >6,000 or mitochondrial transcript proportions >20%. Doublets were removed via DoubletFinder. Data were log-normalized, and dimensionality reduction (PCA and UMAP) was performed. Cell types were annotated using canonical markers. Intercellular communication was inferred using CellChat (v1.6.0), prioritizing ligand–receptor pairs originating from malignant ductal cells.

To interrogate KLF5-centered regulatory logic at single-cell resolution, we performed a network-based virtual knockout using the scTenifoldKnk package (Osorio et al., 2022). A cell-type-specific gene regulatory network (GRN) for the malignant ductal lineage was inferred using the scTenifoldNet framework, which implements principal component regression-based network inference from single-cell expression profiles. Virtual knockout of KLF5 was conducted by computationally removing the KLF5 node (and its associated edges) from the inferred GRN, followed by network re-alignment and stabilization. The perturbation effect was quantified using manifold alignment-based distances and converted to Z-score-normalized perturbation scores to enable gene-wise ranking of downstream effects. Genes were ranked by perturbation Z-scores, and the inferred directionality was interpreted as predicted decreases/increases in regulatory activity (network influence) rather than directly measured differential expression.

### 2.10 Cell culture and DEHP exposure

The human pancreatic cancer cell line PANC-1 was obtained from Wuhan Procell Life Science & Technology Co., Ltd. (Wuhan, China). Cells were maintained in DMEM (HyClone, Shanghai, China) supplemented with 10% FBS (Procell) and 1% penicillin–streptomycin (Solarbio, Beijing, China) at 37 °C in a humidified 5% CO_2_ atmosphere. DEHP (Sigma-Aldrich, St. Louis, MO, United States) was prepared as a concentrated stock solution and diluted in complete medium immediately before use. Cells were treated with DEHP at 0 (control), 10, or 20 μg/mL for 24 h. These concentrations were selected based on commonly used mechanistic *in vitro* exposure ranges for DEHP and our preliminary viability screening, aiming to capture measurable pathway perturbations under sub-cytotoxic conditions (Landkocz et al., 2011). We acknowledge that 10–20 μg/mL exceeds typical general-population biomonitoring levels and therefore represents a high-end *in vitro* exposure used for mechanistic interrogation rather than a direct proxy of average environmental exposure. In particular, these concentrations represent a balance between eliciting detectable molecular/phenotypic responses and maintaining cell viability to interrogate mechanistic perturbations.

### 2.11 Cell viability assay

Cell viability/metabolic activity was measured using a Cell Counting Kit-8 (CCK-8; Beyotime, China). PANC-1 cells were seeded in 96-well plates at 5,000 cells per well. Cells were then exposed to DEHP at the indicated concentrations for 24 h, with the 0 μg/mL group serving as the control. After exposure, the medium was replaced with fresh DEHP-free complete medium, and the post-exposure time course was initiated. At 0, 24, 48, 72, and 96 h after medium replacement, 10 µL of CCK-8 reagent was added to each well and incubated for 2 h in the dark. Absorbance was read at 450 nm (OD450) using a microplate reader.

### 2.12 Quantitative real-time PCR

Total RNA was extracted using TRIzol (Vazyme, China). For each sample, 1 µg RNA was reverse-transcribed using the HiScript III First Strand cDNA Synthesis Kit (Vazyme). Quantitative real-time PCR (qRT-PCR) was performed using ChamQ Universal SYBR qPCR Master Mix (Vazyme) on a LightCycler 480 system (Roche, Shanghai, China). Relative expression was calculated using the 2^−ΔΔCT^ method and normalized to GAPDH. Primer sequences were as follows: MMP7: forward 5′-CAT​GAT​TGG​CTT​TGC​GCG​AG-3′, reverse 5′-AGA​CTG​CTA​CCA​TCC​GTC​CA-3'; KLF5: forward 5′-GTC​AGT​TTC​TTC​CAC​AGC​AGG​C-3′, reverse 5′-GTG​AAT​CGC​CAG​TTT​GGA​AGC​A-3'; GAPDH: forward 5′-CCC​ATG​TTC​GTC​ATG​GGT​GT-3′, reverse 5′-TGG​TCA​TGA​GTC​CTT​CCA​CG-3'.

### 2.13 Western blot analysis

After treatment, cells were lysed in RIPA buffer containing protease inhibitors (Beyotime, China). Protein (20 µg per lane) was separated using 10% SDS-PAGE and transferred to PVDF membranes (Millipore, China). Membranes were blocked with 5% non-fat milk and incubated overnight at 4 °C with antibodies against KLF5 (1:1000; Proteintech, China), MMP7 (1:1000; Proteintech, China), and β-actin (1:5000; Proteintech, China). Bands were detected using an ECL Kit (Vazyme) and quantified in ImageJ.

### 2.14 Wound healing assay

Cell migration was evaluated using a scratch wound assay. PANC-1 cells were grown to confluence in 12-well plates and treated with DEHP at 0, 10, or 20 μg/mL for 24 h. Medium was then replaced with serum-free DMEM containing the corresponding DEHP concentration, and cells were incubated for 12 h to reduce proliferation-related effects. A linear scratch was introduced using a sterile 200-µL pipette tip, and detached cells were removed by washing twice with PBS. Fresh serum-free medium was added, and images were captured at 0, 24, 48, and 72 h using an inverted microscope (Olympus).

### 2.15 Transwell invasion assays

Invasion assays were performed using Transwell chambers (8-µm pore size; Corning, China). Membranes were pre-coated with Matrigel (BD Biosciences, Bedford, MA, United States) according to the manufacturer’s instructions. PANC-1 cells were treated with DEHP at 0, 10, or 20 μg/mL for 24 h. Following treatment, the cells were harvested, and 1 × 10^5^ cells were suspended in serum-free DMEM and seeded into the upper chamber. DMEM containing 10% FBS was added to the lower chamber as a chemoattractant. After 24 h of incubation, non-invading cells on the upper surface were removed. Cells that had invaded the lower surface were fixed with 4% paraformaldehyde and stained with 0.1% crystal violet. Cells were imaged, and five random fields per replicate were counted.

### 2.16 Construction of the adverse outcome pathway framework

The AOP linking DEHP exposure to pancreatic cancer was constructed in accordance with OECD guidelines using a weight-of-evidence approach. Evidence across scales was integrated, including computational modeling, single-cell analyses, and *in vitro* validation. The MIE was proposed as a putative direct interaction between DEHP and KLF5, supported *in silico* by molecular docking and MD simulations, and requiring experimental target-engagement validation. Key events were organized into cellular-level responses (transcriptional dysregulation and phenotypic changes in PANC-1 cells) and tissue-level remodeling of the immune microenvironment. Key event relationships connecting the initiating event to the adverse outcome of pancreatic cancer progression were developed by integrating Mendelian randomization results, regulatory network perturbation analyses, and the supporting literature.

## 3 Results

### 3.1 Identification of DEHP-associated targets in pancreatic cancer

**FIGURE 1:**
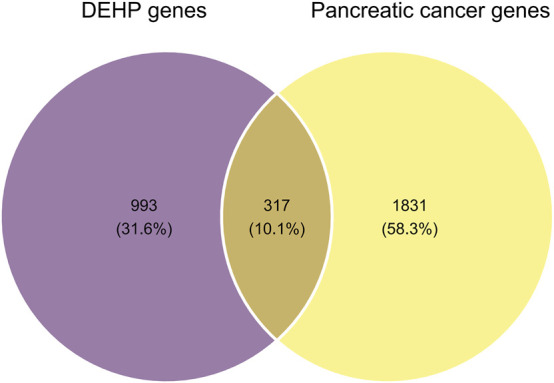
Identification of potential therapeutic targets for DEHP-induced pancreatic cancer. Venn diagram illustrating the intersection analysis between putative DEHP-related targets (n = 1,310, retrieved from ChEMBL, STITCH, and SwissTargetPrediction) and pancreatic cancer-associated genes (n = 2,148, retrieved from GeneCards and OMIM). The overlapping region identifies 317 common targets, which serve as the potential molecular basis for DEHP-induced tumorigenesis.

Database screening retrieved 1,310 putative DEHP targets (Supplementary Table S1) and 2,148 pancreatic cancer-associated genes (Supplementary Table S2). Intersection analysis identified 317 common targets (Supplementary Table S3), representing a candidate molecular interface between DEHP-associated target space and PDAC biology potentially relevant to DEHP-associated pancreatic tumorigenesis (Figure 1).

### 3.2 Functional enrichment and pathway analysis

**FIGURE 2:**
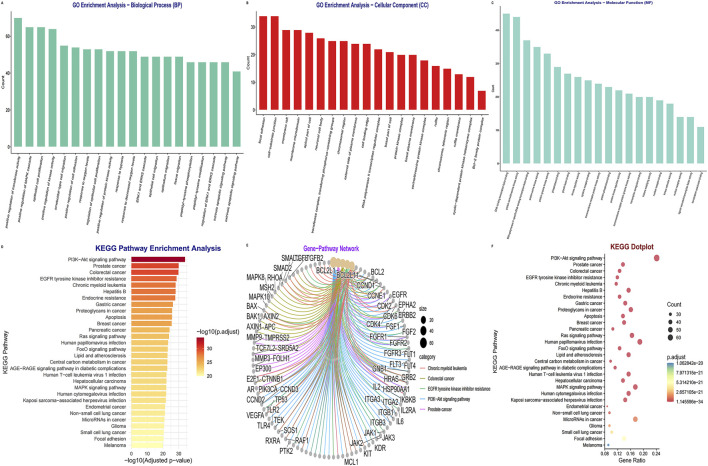
Functional enrichment and pathway analysis of DEHP–pancreatic cancer intersection targets. **(A–C)** Gene Ontology (GO) over-representation analysis for **(A)** biological process (BP), **(B)** cellular component (CC), and **(C)** molecular function (MF). Bars indicate the number of enriched genes annotated to each term (count); significance was assessed using Benjamini–Hochberg FDR (*p*.adjust <0.05). **(D)** KEGG pathway enrichment bar plot. Bar length and color represent −log10(BH-FDR–adjusted P-value), highlighting PI3K–AKT signaling as the top-enriched pathway. **(E)** Gene–pathway crosstalk network for the five most significant KEGG pathways; node size reflects connectivity (degree), emphasizing shared high-connectivity nodes (such as EGFR, ERBB2, and CCND1) across pathways. **(F)** KEGG dot plot showing gene ratio (x-axis), gene count (bubble size), and BH-FDR-adjusted *p*-value (color).

To characterize the functional landscape of the DEHP–PDAC target intersection, we performed GO and KEGG enrichment analyses (Supplementary Tables S4-S5). GO-BP terms were prominently enriched for kinase-driven signaling and growth-related programs, including positive regulation of the MAPK, ERK1, and ERK2 cascades, along with epithelial cell proliferation (Figure 2A). Enriched CC terms localized these gene products to signaling-associated structures such as focal adhesion and membrane raft/microdomain (Figure 2B). MF terms further supported roles in kinase activities (including protein serine/threonine/tyrosine kinase activity) and regulatory interactions such as DNA-binding transcription factor binding (Figure 2C).

KEGG analysis identified the PI3K–AKT signaling pathway as the top-enriched module (Figures 2D,F), together with canonical oncogenic and stress–response pathways (such as MAPK, Ras, FoxO, and apoptosis) and pathways annotated in KEGG as therapy-resistance mechanisms (such as EGFR tyrosine kinase inhibitor resistance). The gene–pathway network (Figure 2E) highlighted shared high-connectivity nodes across these pathways (EGFR/ERBB2/CCND1 and PI3K–AKT/MAPK axis components), consistent with coordinated perturbations in growth factor signaling and downstream survival/proliferation programs.

### 3.3 Screening of core biomarkers via ensemble machine learning

**FIGURE 3:**
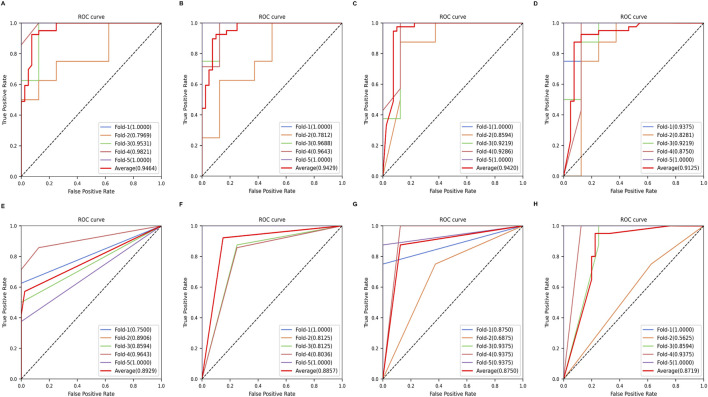
Predictive performance of the multi-algorithm machine-learning framework. ROC curves were generated using five-fold cross-validation. Thin lines represent ROC curves from individual folds, and the thick red line denotes the mean ROC curve (average AUC shown in the legend). The diagonal dashed line indicates the no-discrimination baseline. Panels: **(A)** XGBoost, **(B)** random forest, **(C)** TabPFN, **(D)** SVM, **(E)** Gaussian process (GPR), **(F)** naive Bayes, **(G)** logistic regression, and **(H)** gradient boosting decision tree (GBDT).

To construct a parsimonious and biologically relevant feature set, we mapped the 317 DEHP–PDAC intersection targets to the GSE15471 expression matrix and retained 26 candidates represented by reliable measurements and sufficient variability (Supplementary Table S6). Using these candidates, we trained eight distinct classifiers within the heterogeneous ensemble framework and evaluated performance using (stratified) five-fold cross-validation on held-out folds (Figures 3A–H). The ensemble demonstrated robust discriminative ability across architectures; XGBoost achieved the highest mean cross-validated AUC (0.946), followed by random forest (AUC = 0.943) and the transformer-based TabPFN (AUC = 0.942).

To mitigate algorithmic bias and idiosyncrasies and derive a stable molecular signature, we applied the pre-specified frequency-based consensus criterion (Section 2.4), yielding six core biomarkers (HK2, ACVR1, KLF5, PDGFRB, ITGA3, and MMP7) prioritized as top-ranked features by at least seven of the eight algorithms.

### 3.4 Feature importance via SHAP analysis

**FIGURE 4:**
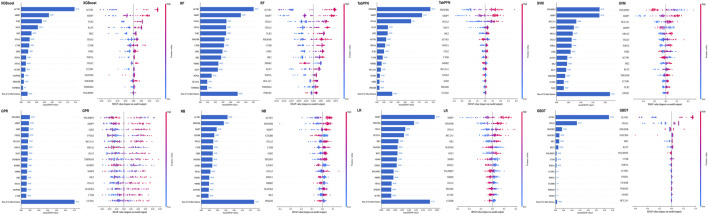
SHAP-based model interpretability across the eight classifiers. For each algorithm, the left panel shows global feature importance quantified via the mean absolute SHAP value (top features displayed; remaining features aggregated as “Sum of other features”), and the right panel shows the SHAP summary (beeswarm) plot. Each dot represents one sample; color indicates the feature value (high to low), and the x-axis shows the SHAP value (impact on the model output; positive values push the prediction toward the pancreatic cancer class). Across models, tree-based classifiers prominently prioritized ACVR1 and MMP7, whereas PDGFRB and ITGA3 were more influential in TabPFN/LR; HK2 and KLF5 showed recurrent, moderate contributions across multiple algorithms.

We used SHAP analysis to interpret model behavior and contextualize the contribution of each candidate gene (Figure 4). The summary beeswarm plot visualized how variations in gene expression influenced the model’s classification output (tumor versus normal). In the XGBoost model, ACVR1 and MMP7 exhibited the strongest contributions to classification. Across the ensemble, KLF5, HK2, and ITGA3 maintained consistently elevated SHAP values across distinct model classes. This cross-model consistency supports their prioritization as stable features within the DEHP–PDAC candidate program rather than algorithm-specific artifacts.

### 3.5 Immune microenvironment characterization

**FIGURE 5:**
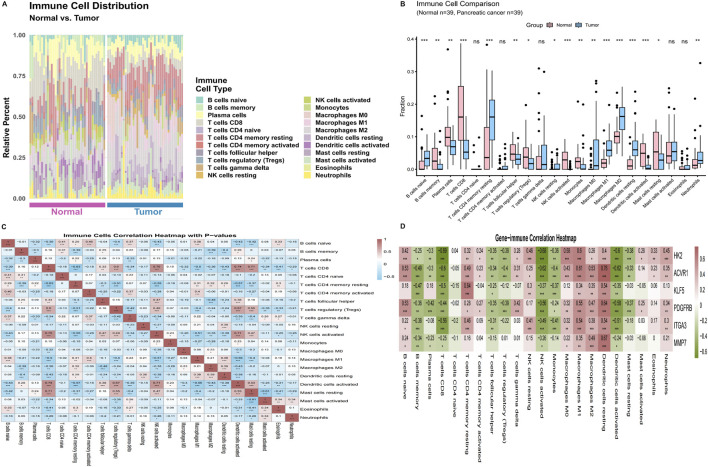
CIBERSORT-inferred tumor immune microenvironment and core gene–immune associations. **(A)** Stacked bar plot showing relative fractions of 22 LM22 immune cell subsets in normal (pink) and tumor (blue) samples. Only samples with CIBERSORT deconvolution *p* < 0.05 were retained for downstream analyses (n = 78; 39 normal and 39 tumor). **(B)** Between-group comparison of immune cell fractions using the Wilcoxon rank-sum test (ns, not significant; *p < 0.05; **p < 0.01; ***p < 0.001), highlighting decreased plasma/memory B-cell components and increased macrophage subsets in tumors. **(C)** Spearman correlation matrix among immune cell subsets (red, positive; blue, negative). **(D)** Spearman correlations between the six-gene core signature and immune cell fractions; colors indicate correlation coefficients (red, positive; green, negative), and asterisks denote statistical significance.

To examine whether the DEHP-associated core program aligns with immune contexture, we deconvoluted 22 immune cell subsets using CIBERSORT (Figure 5A). Compared with normal tissues, PDAC samples exhibited marked immune heterogeneity (Figure 5B), including reduced humoral components (plasma cells and memory B cells) and a shift toward myeloid lineages, with increased macrophage subsets (M0/M1/M2). The overall fraction of CD8^+^ T cells was lower in tumor samples. Immune–immune correlation analysis revealed coordinated patterns among infiltrates (Figure 5C); for example, CD8^+^ T cells were positively correlated with activated NK cells and inversely correlated with resting memory CD4^+^ T cells.

Gene–immune association analysis (Figure 5D) showed that core genes, including KLF5 and ITGA3, were inversely associated with inferred CD8^+^ T-cell infiltration. In contrast, PDGFRB was positively associated with M2 macrophages, consistent with a stromal-/myeloid-skewed immunosuppressive milieu. Collectively, these results link the DEHP-related core signature to immune features characterized by reduced cytotoxic infiltration and enhanced myeloid/stromal components, while recognizing that CIBERSORT deconvolution and correlation analyses are inferential and should be interpreted cautiously.

### 3.6 Clinical prognostic validation

**FIGURE 6:**
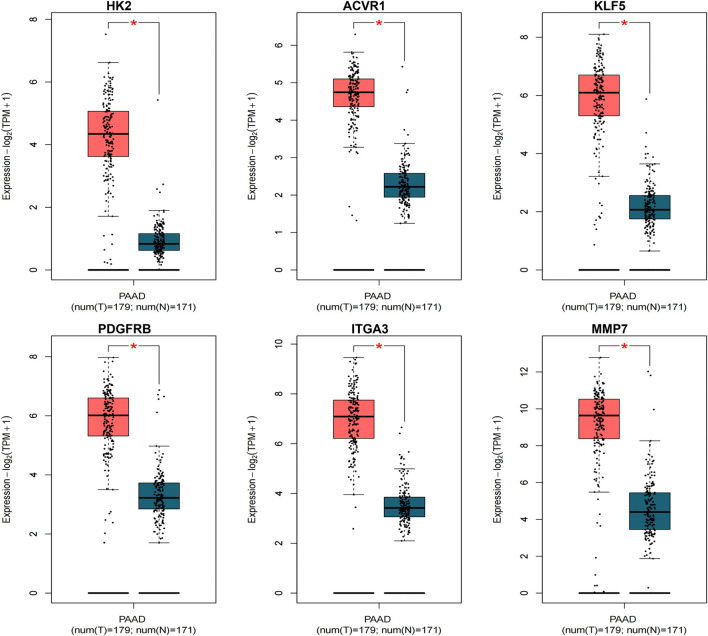
Differential expression validation of the six-gene signature in TCGA-PAAD and GTEx. Box plots show mRNA expression levels (log_2_ [TPM +1]) of HK2, ACVR1, KLF5, PDGFRB, ITGA3, and MMP7 in TCGA-PAAD tumors (red, n = 179) versus GTEx normal pancreas tissues (blue, n = 171) from the UCSC Xena Toil dataset. *p*-values were calculated using a two-sided Wilcoxon rank-sum test (*p < 0.05).

**FIGURE 7:**
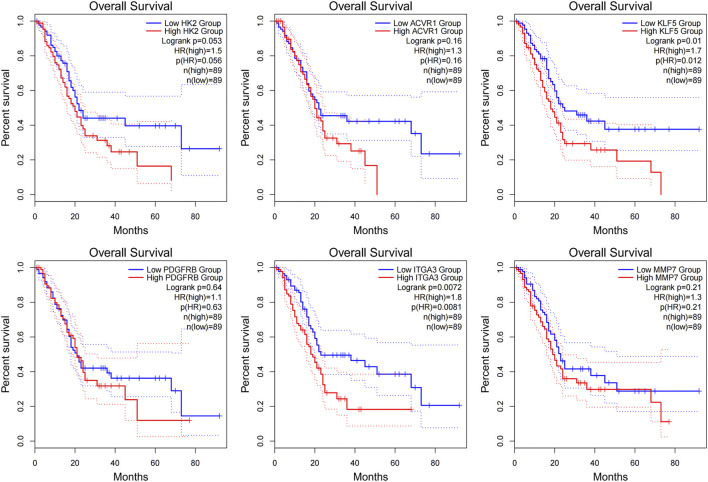
Kaplan–Meier overall survival analyses stratified by gene expression in TCGA–PAAD. Patients were dichotomized into high- (red) and low-expression (blue) groups using the median expression of each gene. Log-rank tests were used to compare survival distributions, and hazard ratios (HR_high) were estimated using univariate Cox models. Dotted lines indicate 95% confidence intervals. High expression of KLF5 (*p* = 0.01) and ITGA3 (*p* = 0.0072) was significantly associated with poorer overall survival, whereas HK2 showed a borderline trend (*p* = 0.053).

External validation in the TCGA–PAAD/GTEx Toil cohort confirmed that all six candidates (HK2, ACVR1, KLF5, PDGFRB, ITGA3, and MMP7) were significantly upregulated in tumor tissues compared with those in normal pancreas tissue (Figure 6). Kaplan–Meier analyses further stratified their prognostic relevance (Figure 7). HK2 showed a borderline association with overall survival (log-rank *p* = 0.053), whereas high expressions of KLF5 (log-rank *p* = 0.01; HR_high = 1.7) and ITGA3 (log-rank *p* = 0.0072; HR_high = 1.8) were significantly associated with poorer survival. These results prioritize KLF5 and ITGA3 as clinically relevant candidates for downstream mechanistic interpretation and further validation.

### 3.7 Causal inference via Mendelian randomization

**FIGURE 8:**
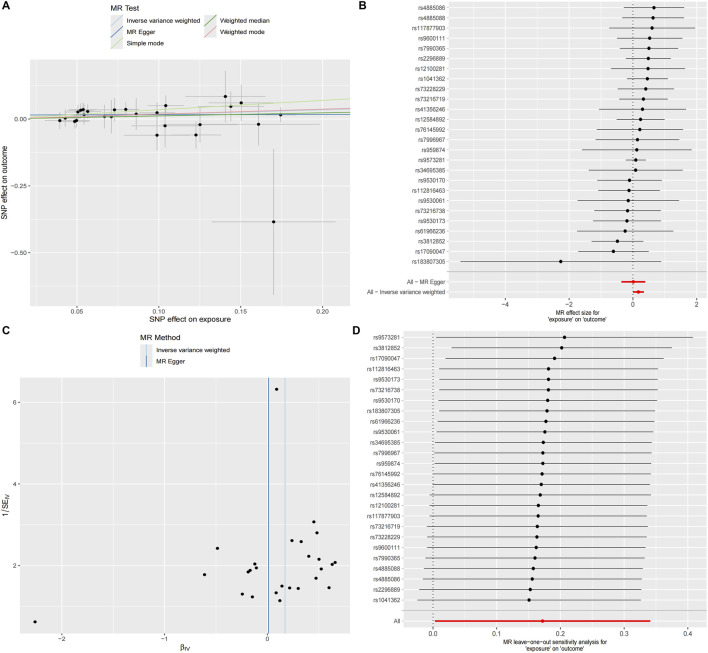
Causal association between KLF5 expression and pancreatic cancer risks inferred by Mendelian randomization (MR). **(A)** Scatter plot of the SNP effects on KLF5 exposure (x-axis) versus pancreatic cancer outcome (y-axis). The slope of the lines corresponds to the causal estimate using different MR methods. **(B)** Forest plot of the causal effects estimated by individual SNPs and the pooled result (red line). **(C)** Funnel plot assessing the symmetry of the causal estimates to detect potential publication bias or heterogeneity. **(D)** Leave-one-out sensitivity analysis demonstrating the stability of the causal effect when excluding individual SNPs. The red line represents the overall IVW estimate.

We performed two-sample MR to evaluate potential causal associations between prioritized candidates and pancreatic cancer risks (Supplementary Table S7). For KLF5, 26 independent cis-eQTL instruments (nSNP = 26) were selected after LD clumping. The IVW method revealed suggestive evidence that genetically predicted KLF5 is associated with an increased risk of pancreatic cancer (OR = 1.188, 95% CI = 1.003–1.406, *p* = 0.046). We acknowledge the borderline significance and have provided detailed instrument strength (F-statistics) in Supplementary Table S8 to support the robustness of these preliminary causal inferences. Sensitivity analyses indicated no significant heterogeneity (Cochran’s Q test, *p* = 0.952) and no evidence of directional horizontal pleiotropy (MR–Egger intercept *p* = 0.351; MR–PRESSO global test *p* = 0.196). Scatter, forest, and funnel plots are provided for visual assessment (Figures 8A–C), and leave-one-out analysis suggested that the IVW estimate was not driven by a single instrument (Figure 8D).

In contrast, no significant causal association was observed for ITGA3 (IVW *p* = 0.240; Supplementary Table S9). Given the modest effect size and borderline significance for KLF5, these MR findings are best interpreted as supportive prioritization rather than definitive causal confirmation.

### 3.8 Molecular docking analysis

**FIGURE 9:**
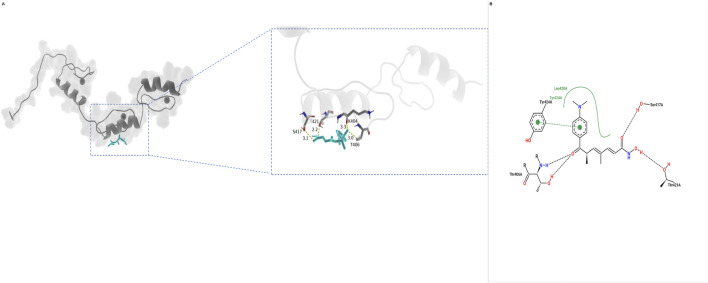
Structural basis of the DEHP–KLF5 interaction revealed by molecular docking. **(A)** Ribbon/surface view showing DEHP (cyan sticks) accommodated in a putative pocket of KLF5. **(B)** 2D interaction map of the binding pose. Dashed lines denote hydrogen bonds/polar contacts (distances in Å), green arcs indicate hydrophobic contacts, and the dotted green line denotes a π–π interaction with Tyr424.

Docking suggested that DEHP can be accommodated in a putative pocket of KLF5 (Figure 9A) based on an experimentally determined KLF5 structure retrieved from the Protein Data Bank (PDB; PDB ID: 2EBT), yielding a Vina affinity score of −6.4 kcal/mol. A representative conformer from the NMR ensemble was selected for structure-based modeling following standard protein preparation (removal of non-protein molecules, addition of hydrogens, and assignment of protonation states). The binding pose involved polar contacts/hydrogen bonds with Thr406 and Ser417 (≈2.2–3.1 Å) and an additional polar contact with Thr421/Lys404 (≈3.3 Å) (Figure 9B). Hydrophobic contacts with Leu420 and a π–π interaction with Tyr424 further stabilized the aromatic phthalate moiety (Figure 9B). Collectively, these interactions support the structural feasibility of a non-covalent DEHP–KLF5 complex within the applied docking framework, suggesting a plausible binding mode in the modeled system. Residue numbering is reported consistently with the reference sequence used for structure preparation, and any differences between PDB and UniProt residue indices should be considered when mapping interaction residues across figures and analyses.

### 3.9 Molecular dynamic stability and conformational landscape of the DEHP–KLF5 complex

**FIGURE 10:**
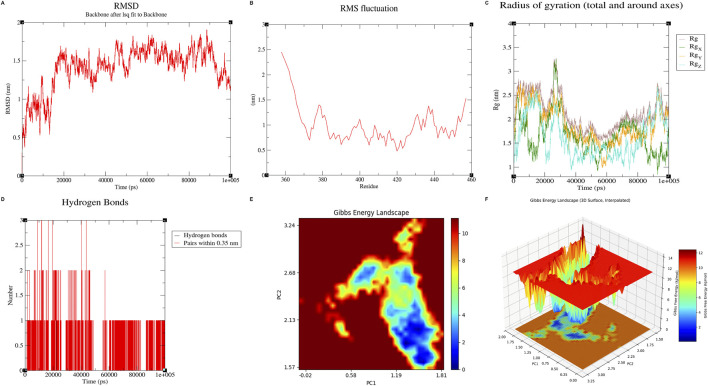
Conformational dynamics of the DEHP–KLF5 complex over a 100-ns MD simulation. **(A)** Protein backbone RMSD (after least-squares alignment to the backbone) showing an initial relaxation followed by fluctuations around an equilibrated range, reflecting structural adaptation. **(B)** Per-residue RMSF demonstrating spatially heterogeneous flexibility within the simulated KLF5 segment; note the elevated mobility at the disordered segment ends (residues ∼355–370 and ∼435–460) contrasted by comparatively restrained fluctuations across the central putative binding region (residues ∼385–425). **(C)** Radius of gyration (Rg) indicating time-dependent compaction/expansion of the complex. **(D)** Time evolution of protein–ligand hydrogen bonds, showing intermittent polar interactions. **(E,F)** Free-energy landscape projected onto PC1 and PC2 (2D contour and 3D surface), where low-energy basins represent preferential conformational states sampled during the trajectory.

To evaluate conformational evolution under explicit–solvent conditions, we performed a 100-ns MD simulation of the DEHP–KLF5 complex. As shown in Figure 10A, the protein backbone RMSD exhibited marked fluctuations and equilibrated in the range of 1.2–1.8 nm, indicating substantial global rearrangement within the simulated segment. Residue-level flexibility profiling (RMSF, Figure 10B) showed that this motion was spatially heterogeneous and was driven primarily by a hyper-flexible region (residues ∼355–375), where fluctuations exceeded 2.0 nm.

In contrast, the putative DEHP-binding region (residues ∼390–440) displayed comparatively restrained fluctuations, suggesting a locally more stable core embedded within a globally dynamic scaffold. The Rg value varied over time (Figure 10C), consistent with breathing motions rather than loss of overall compactness or disassembly of the binding interface.

Despite global dynamics, interaction metrics indicated persistent proximity without complete dissociation over the 100-ns window. Hydrogen bonds were intermittent (Figure 10D), consistent with a binding mode in which hydrophobic packing contributes substantially. Contact analysis showed an early increase in the number of protein–ligand atom pairs within 0.35 nm that remained elevated thereafter (Supplementary Figure S1), and the minimum protein–ligand distance remained in a close-contact regime (∼0.20–0.22 nm) with reduced fluctuations after equilibration (Supplementary Figure S2). The free-energy landscape (FEL) identified discrete low-energy basins (Figures 10E,F), indicating preferred metastable states sampled during the trajectory. Collectively, these results are consistent with a dynamic adaptation model in which DEHP maintains persistent interfacial engagement within the modeled system.

### 3.10 Single-cell resolution and *in silico* perturbation reveal KLF5-linked remodeling programs

**FIGURE 11:**
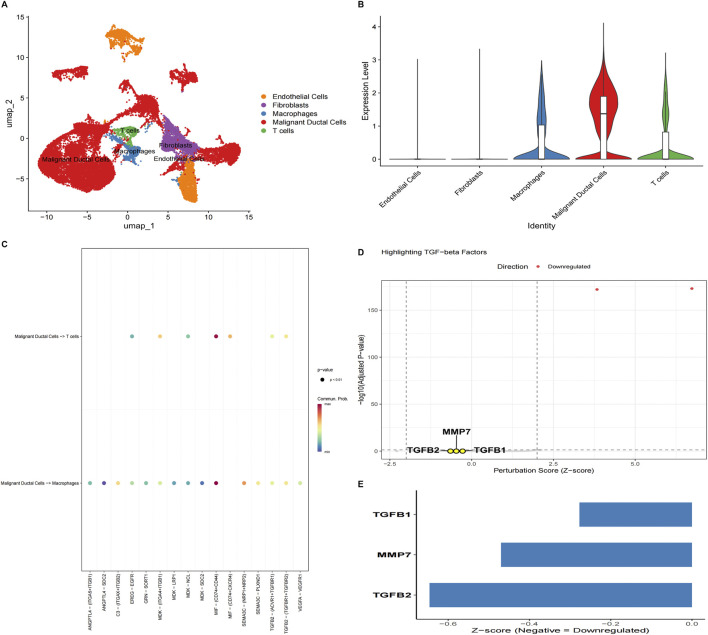
Single-cell profiling and network-based virtual knockout implicate KLF5-associated microenvironmental signaling in PDAC. **(A)** UMAP of GSE291124 PDAC tumors annotated into five major lineages. **(B)** Violin plots of KLF5 expression across lineages, showing predominant expression in malignant ductal cells with lower but detectable signals in immune subsets. **(C)** CellChat bubble plot of significant ligand–receptor interactions originating from malignant ductal cells targeting macrophages and T cells; color denotes communication probability, and dot size reflects statistical significance. **(D)** Volcano-style plot summarizing virtual knockout perturbation results (perturbation Z-score vs. −log10 adjusted *p*-value); highlighted genes (TGFB1, TGFB2, and MMP7) are shown for the TGF-β axis. Directionality indicates predicted decreases in regulatory activity (not directly measured expression). **(E)** Predicted activity changes (Z-scores) for TGFB1, TGFB2, and MMP7 in the KLF5 virtual KO state; negative values indicate reduced predicted activity, providing hypothesis-generating support for a KLF5-linked TGF-β/MMP7 program. These perturbation scores reflect computational predictions within an inferred network and should be interpreted as hypothesis-generating support rather than directly measured differential expression.

To define the cellular origin of KLF5 expression and its potential relationship with microenvironmental signaling, we analyzed scRNA-seq data from four PDAC tumors (Supplementary Tables S10–S12). UMAP resolved five major cellular lineages (Figure 11A). KLF5 expression was highest in malignant ductal cells, with comparatively lower but detectable expression in immune compartments (Figure 11B), supporting a predominantly tumor-intrinsic pattern. CellChat analysis inferred prominent outgoing communication from malignant cells involving MIF-(CD74^+^CD44) and TGF-β pathways (particularly TGFB2–TGFBR1/2) (Figure 11C).

We next performed a virtual knockout analysis in malignant ductal cells to interrogate KLF5-centered regulatory logic. Using a gene regulatory network-based perturbation framework, we simulated deletion of KLF5 and computed gene-level perturbation scores (Z-score-normalized distances) to quantify predicted transcriptional consequences (full results in Supplementary Table S13). *In silico* deletion of KLF5 produced the largest perturbation score within the inferred network (Figure 11D). For the downstream TGF-β axis, TGFB1, TGFB2, and MMP7 showed modest perturbation magnitudes (Figure 11E). Directionality analysis indicated a consistent negative trend in predicted activity for all three genes.

Although these changes did not reach conventional thresholds for statistical significance, the shared direction is compatible with a model in which KLF5 contributes to sustaining a TGF-β-enriched, matrix-remodeling program in malignant ductal cells. Accordingly, the virtual KO results are interpreted as hypothesis-generating support that motivates experimental probing of KLF5 dependency.

### 3.11 Experimental validation: DEHP promotes malignant phenotypes through induction of the KLF5/MMP7 axis

**FIGURE 12:**
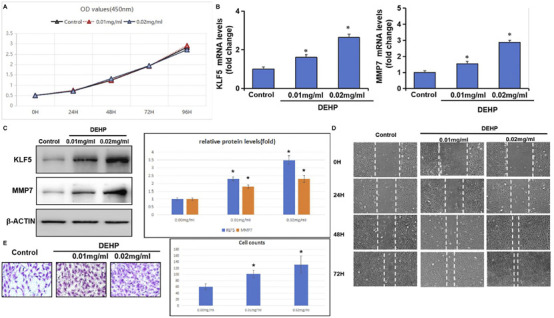
Experimental validation of DEHP-induced KLF5/MMP7 expression and malignant phenotypes in PANC-1 cells. **(A)** CCK-8 assay showing OD450 at 0, 24, 48, 72, and 96 h following 24 h of DEHP treatment (0, 10, or 20 μg/mL) and subsequent replacement with DEHP-free complete medium. **(B)** qRT-PCR analysis of KLF5 and MMP7 mRNA after 24 h treatment with DEHP (0, 10, or 20 μg/mL); transcript levels were normalized to GAPDH and expressed relative to the control group. **(C)** Representative Western blots of KLF5 and MMP7 with β-actin as a loading control, with densitometric quantification presented as fold change relative to the control (set to 1.0). **(D)**Wound-healing assay showing representative images at 0, 24, 48, and 72 h following DEHP treatment (0, 10, or 20 μg/mL). **(E)** Transwell invasion assay showing representative crystal violet-stained images and quantification of invaded cell numbers (cells counted in five random fields per replicate) after DEHP exposure (0, 10, or 20 μg/mL). Data are presented as the mean ± SD from three independent experiments. Statistical significance was assessed using one-way ANOVA with Dunnett’s post hoc test versus control (*p < 0.05).

To experimentally assess whether DEHP exposure is sufficient to induce the KLF5/MMP7 module and shift tumor cells toward a more aggressive phenotype, we profiled molecular and functional responses in PANC-1 cells. To minimize confounding by overt cytotoxicity, we first evaluated cell viability. Across a 96-h window, DEHP exposure (10 and 20 μg/mL, equivalent to 0.01 and 0.02 mg/mL, respectively) produced no marked reduction in metabolic activity relative to controls (Figure 12A), supporting the use of these concentrations for downstream assays.

After 24 h of exposure, qRT-PCR and Western blotting showed dose-dependent upregulation of KLF5 and MMP7. DEHP treatment at 20 μg/mL induced an ≈3.5-fold increase in KLF5 protein abundance and an ≈2-fold increase in MMP7 levels relative to controls (Figures 12B,C).

Functionally, DEHP-treated monolayers showed faster wound closure than controls (Figure 12D). Transwell assays further indicated increased invasive capacity, with an approximately two-fold increase in invaded cells at the highest concentration (Figure 12E). Collectively, these data provide experimental support that DEHP exposure is associated with the concomitant induction of the KLF5/MMP7 module and enhanced motility in PANC-1 cells. We characterize these findings as a cellular-level key-event readout aligned with our computational model, rather than a definitive validation of the entire axis, as the strict functional dependency of KLF5 for these phenotypes remains to be tested via targeted perturbation.

### 3.12 Construction of the adverse outcome pathway

**FIGURE 13:**
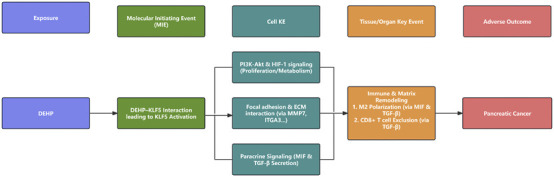
Proposed AOP for DEHP-induced pancreatic cancer. This schematic summarizes an OECD-aligned working AOP linking DEHP exposure to PDAC progression across molecular, cellular, and tissue scales. A putative, *in silico*-supported DEHP–KLF5 interaction (docking/MD) is proposed as a candidate MIE, followed by tumor-intrinsic transcriptional dysregulation consistent with activation of PI3K–AKT/HIF-1 programs and ECM-related effectors (such as MMP7/ITGA3), together with inferred malignant-cell paracrine signaling involving TGF-β and MIF. These signals are hypothesized to contribute to TIME remodeling, including stromal activation/fibrosis, M2 macrophage enrichment, and reduced effective CD8^+^ T-cell infiltration, thereby facilitating the adverse outcome of pancreatic cancer progression. The DEHP–KLF5 interaction is presented as a putative, *in silico*-supported candidate MIE requiring direct target-engagement validation; TIME-related KEs are inferred and should be experimentally tested in multicellular models.

Finally, we integrated computational predictions with *in vitro* validation to assemble a refined working AOP linking DEHP exposure to pancreatic cancer progression (Figure 13). The pathway is initiated by environmental exposure to DEHP. Docking and MD simulations support the *in silico* plausibility of an interaction between DEHP and KLF5 as a putative MIE within the modeled system, while direct target engagement remains to be established experimentally. In parallel, DEHP exposure increased KLF5 protein abundance in PANC-1 cells, consistent with the activation of a KLF5-centered program. Sustained KLF5 activity is then proposed to propagate downstream transcriptional dysregulation, including the validated effector MMP7, leading to cellular-level KEs characterized by metabolic reprogramming (HK2) and enhanced migration/invasion (MMP7/ITGA3).

These tumor-intrinsic perturbations are associated with tissue-level hypotheses through inferred remodeling of the TIME, integrating deconvolution and single-cell communication analyses that were consistent with malignant-cell-derived MIF and TGF-β signaling. Within the AOP schematic, these inferred tissue-level KEs include stromal activation/fibrosis, M2 macrophage enrichment, and reduced effective CD8^+^ T-cell infiltration, which together provide a plausible microenvironmental context for PDAC progression. Where immune and stromal states were not directly measured, the corresponding KEs are presented as inference-based components intended to guide targeted validation. These inference-based TIME features are directionally consistent with prior PDAC single-cell atlases reporting myeloid-enriched, T-cell-limited ecosystems and pronounced stromal programs (Peng et al., 2019; Elyada et al., 2019), along with reviews describing robust immune-exclusion barriers in PDAC (Hessmann et al., 2020).

## 4 Discussion

### 4.1 Bridging environmental exposure and pancreatic tumorigenesis through a multi-scale systems toxicology framework

Pancreatic cancer reflects the combined effects of inherited susceptibility and acquired exposures (Peduzzi et al., 2025), and endocrine-disrupting chemicals (EDCs) have increasingly been considered part of the exposure landscape relevant to pancreatic tumorigenesis. DEHP is a high-use plasticizer and a well-recognized endocrine disruptor, yet specific mechanistic links between DEHP exposure and pancreatic carcinogenesis remain insufficiently resolved. We integrated network toxicology, heterogeneous ensemble machine learning, MR, molecular docking and MD, single-cell transcriptomics, and *in vitro* functional assays to assemble a working AOP connecting DEHP exposure to PDAC-relevant molecular and cellular programs.

Rather than positioning DEHP as a nonspecific oxidative stressor, our results are more consistent with preferential engagement of a KLF5-centered regulatory axis. Multiple, independent lines of evidence converged on KLF5 across Sections 3.3–3.11: it was reproducibly prioritized by the ensemble framework, associated with adverse prognosis in TCGA–PAAD, showed suggestive genetic support for pancreatic cancer risks in MR, was induced by DEHP in PANC-1 cells, and aligned with a tumor-intrinsic role compatible with a TGF–β/MMP7-linked matrix-remodeling and immunomodulatory program inferred from single-cell and network analyses (Krstic and Santibanez, 2014; Diaferia et al., 2016). Taken together, these observations support a systems-level rationale for considering KLF5 as a mechanistic bridge between exposure and tumor-promoting biology while noting that several links—particularly direct target engagement and tissue-level immune consequences—remain inference-based at this stage.

### 4.2 Consensus biomarker identification and genetic support for KLF5

A recurring challenge in toxicogenomics is distinguishing stable biomarkers from high-dimensional noise. By combining a heterogeneous ensemble strategy with a stringent frequency-based consensus filter across eight algorithms, we defined a reproducible six-gene signature (HK2, ACVR1, KLF5, PDGFRB, ITGA3, and MMP7) prioritized across model classes with distinct inductive biases. This consensus design reduces reliance on any single algorithm and emphasizes features that remain informative across heterogeneous architectures.

Clinical and genetic analyses further sharpened this signature. In TCGA–PAAD, all six genes were upregulated in tumor tissues relative to normal pancreas, whereas KLF5 and ITGA3 were additionally associated with worse overall survival (He et al., 2018; Li et al., 2025), suggesting that they capture prognostically meaningful variation beyond differential expression alone. Two-sample MR provided orthogonal support for prioritizing KLF5: genetically predicted higher KLF5 expression provided suggestive evidence for an association with an increased pancreatic cancer risk (OR = 1.188, *p* = 0.046), with no evidence of substantial heterogeneity or directional pleiotropy in sensitivity analyses. Given the modest effect size and borderline significance, MR is best considered supportive prioritization rather than definitive causal proof, and residual context dependence (including tissue specificity and context specificity of the eQTL instruments) cannot be excluded.

### 4.3 Molecular initiating event: structural plausibility of DEHP–KLF5 binding

A credible AOP benefits from a clearly articulated molecular initiating event. Our docking and MD analyses suggest that DEHP can be accommodated within a putative hydrophobic pocket of KLF5 using an experimentally determined KLF5 structure retrieved from the Protein Data Bank (PDB; PDB ID: 2EBT), with an affinity of approximately −6.4 kcal/mol. The predicted binding pose was supported by hydrogen bonds/polar contacts with Thr406 and Ser417 and an additional polar contact with Thr421/Lys404, together with hydrophobic interactions involving Leu420 and a π–π interaction with Tyr424. Across the 100-ns trajectory, DEHP remained resident within the pocket, while the protein backbone exhibited global flexibility with comparatively restrained fluctuations around the proposed binding region, suggesting a locally stable core embedded within a dynamic scaffold.

Interaction metrics and the conformational landscape supported this view. Protein–ligand atom pairs within 0.35 nm increased early in the simulation and remained persistently elevated, the minimum protein–ligand distance stayed in a close-contact regime (∼0.20–0.22 nm) with reduced fluctuations after equilibration, and the free-energy landscape indicated that the complex preferentially sampled a limited number of low-energy basins, consistent with metastable conformations during the trajectory.

These structure-based results should nevertheless be interpreted as hypothesis-generating. Although the analyses were grounded in an experimentally resolved PDB structure, the available structural coverage represents only a defined region/domain of KLF5 and does not capture the full-length transcription factor in its native cellular or chromatin-bound context. Consequently, this choice constitutes a limitation based on existing structural data as domain-level simulations may not fully reflect the full-length conformational or regulatory context. Accordingly, the inferred pocket, binding pose, and dynamics should be interpreted as properties of the resolved structural region used for modeling and should not be overextended to full-length KLF5 regulation without direct target-engagement validation in living systems (Wang et al., 2021). Together, these observations motivate a working “two-hit” framework in which DEHP exposure could (i) engage KLF5 through a structurally plausible interaction and (ii) coincide with pro-survival signaling modules (such as PI3K–AKT) that modulate KLF5 stability or activity (Chen et al., 2023). Direct target-engagement and functional dependency assays (such as CETSA/DARTS, SPR, and cell-based reporter systems) are required to determine whether DEHP binding measurably affects the half-life, ubiquitination, or transcriptional output of KLF5 in living cells.

### 4.4 KLF5-centered regulatory logic and microenvironmental remodeling

A key mechanistic question is how a tumor-intrinsic KLF5-centered perturbation could extend to microenvironmental remodeling. Bulk immune deconvolution suggested a TIME biased toward myeloid/stromal features, including enrichment of macrophage populations, reduced humoral components, and a lower CD8^+^ T-cell fraction in tumor tissues. Within tumors, higher KLF5 expression was inversely associated with inferred CD8^+^ T-cell infiltration, consistent with an immune-exclusion-like pattern in KLF5-high contexts. In parallel, PDGFRB expression positively correlated with M2 macrophages (and mast cell-related subsets in some analyses), supporting the emergence of a fibroblast/pericyte-linked, stromal-/myeloid-skewed immunosuppressive milieu. Conceptually, these findings reflect two complementary views: CIBERSORT highlights tumor-versus-normal shifts in overall immune composition, whereas gene–immune correlations capture within-tumor variation, suggesting that KLF5-high programs may mark locally less cytotoxic niches even when bulk immune content varies across patients.

Single-cell analyses refined these inferences. KLF5 expression was largely localized to malignant ductal cells, supporting a predominantly tumor-intrinsic regulator rather than an immune or stromal marker. CellChat analysis inferred prominent outgoing communication from malignant ductal cells involving MIF–(CD74^+^CD44) and TGF-β pathways (particularly TGFB2–TGFBR1/2) toward macrophages and T cells, implicating malignant cells as potential initiators of immunomodulatory cues within the tumor ecosystem (Li et al., 2020). Network-based virtual knockout further provided hypothesis-generating context: *in silico* KLF5 deletion produced the largest perturbation score within the inferred network, and TGFB1, TGFB2, and MMP7 showed modest but consistently negative predicted activity changes (Zhou X. et al., 2025). Although the magnitudes were small—plausibly reflecting regulatory redundancy and conservative thresholds—the shared direction is compatible with a model in which KLF5 contributes to sustaining a TGF-β-enriched, matrix-remodeling program in malignant ductal cells.

Overall, the convergent directionality across bulk, single-cell, and network views supports a coherent working model in which KLF5-high tumor cells align with MMP7 expression and TGF-β/MIF-associated crosstalk, which may favor stromal activation and immune attenuation (Ma et al., 2020). This inference is directionally consistent with PDAC single-cell atlases and microenvironment reviews that report myeloid-enriched, T-cell-limited ecosystems and prominent stromal programs while noting that spatial/functional validation is required.

### 4.5 *In vitro* validation of the DEHP–KLF5–MMP7 axis and linkage to adverse outcomes

We next asked whether DEHP exposure is sufficient to activate the KLF5–MMP7 module and shift PDAC cells toward a more aggressive phenotype. In PANC-1 cells, short-term DEHP treatment (10–20 μg/mL) increased KLF5 and MMP7 at both mRNA and protein levels, accompanied by enhanced migration and invasion. These data provide a tumor-intrinsic, cellular-level key-event readout consistent with engagement of an MMP7-linked matrix-remodeling program.

These experiments do not establish strict KLF5 dependency for each phenotype; defining dependency requires targeted perturbation (such as KLF5 knockdown/overexpression with rescue) and assessment of whether DEHP-induced motility and invasion are abolished or attenuated. Nevertheless, the observed directionality was consistent with the logic of the virtual knockout (predicted dampening of MMP7/TGF-β axis activity upon KLF5 deletion) and supports the plausibility of the DEHP–KLF5–MMP7 branch within the proposed AOP. At the tissue level, integrating MMP7 with inferred malignant-cell signaling via TGF-β and MIF provides a mechanistically coherent bridge to stromal remodeling and immune attenuation hypotheses (Fey et al., 2024; Wang et al., 2025). Because drug response was not directly assayed, the linkage to therapy tolerance is framed as a pathway-focused rationale (such as PI3K–AKT-coupled survival signaling and TGF-β-linked stromal remodeling frequently observed in therapy-tolerant niches) rather than a demonstrated resistance phenotype (Zhang and Zhang, 2020). From an AOP perspective, coordinated induction of KLF5/MMP7 and the associated motility/invasion phenotypes offers tractable, quantitative cellular-level KEs that can support AOP-informed testing and weight-of-evidence evaluation for DEHP and related plasticizers.

### 4.6 Limitations and future directions

Several limitations should be considered when interpreting this working AOP. First, the proposed DEHP–KLF5 interaction is supported by docking/MD plausibility and indirect cellular responses, rather than direct target-engagement measurements. Biophysical and engagement assays (CETSA/DARTS, SPR, and isothermal titration calorimetry) are important to confirm binding, define the relevant structural region in full-length KLF5, and quantify kinetics under physiologically relevant conditions. Second, although *in vitro* assays and network-based inferences consistently support the induction of a KLF5–MMP7 module, dependency should be tested by genetic perturbation with rescue and validated in more physiologically relevant systems. Furthermore, our current experimental validation relied on a single pancreatic cancer cell line (PANC-1). Although PANC-1 was selected for its prominent mesenchymal-like features—which are highly congruent with the DEHP-induced ECM remodeling and TGF-β axis activation identified in our computational models—we acknowledge that a single line may not fully reflect the profound inter-tumoral heterogeneity of PDAC. Testing across a broader panel of cell lines and patient-derived organoids is necessary to ensure the generalizability of the KLF5-centered mechanism. MR inference also depends on the tissue context; thus, the KLF5 MR signal should be interpreted as suggestive and ideally replicated using pancreas- or tumor-relevant eQTL resources. Third, the microenvironmental component is largely inference-based. Deconvolution and CellChat infer cellular composition and communication, and the scRNA-seq analysis was limited to four tumors. We experimentally validated only tumor-intrinsic effects (KLF5/MMP7 induction and increased migration/invasion) and did not directly quantify macrophage polarization, stromal activation, or spatial T-cell distribution. Finally, regarding exposure relevance, although the doses of 10 and 20 μg/mL are within the range used for investigating phthalate-induced carcinogenesis *in vitro*, we acknowledge that extrapolating these acute, high-dose responses to chronic, low-level human exposure requires caution. Notably, although these concentrations exceed general-population biomonitoring levels, using slightly elevated doses to observe signal transduction alterations under acute chemical stress is a common practice in mechanistic toxicology. Future studies incorporating pharmacokinetic modeling are essential to translate these findings into quantitative human risk assessments.

Despite these constraints, combined evidence from ensemble learning, MR, structural modeling, single-cell/network analyses, and *in vitro* assays supports a coherent working model in which KLF5 and a downstream TGF-β/MMP7-linked program may act as a conduit for DEHP-associated PDAC progression. Integration with exposure assessment and pharmacokinetic modeling is important for translating this qualitative, mechanism-informed AOP into more quantitative, decision-relevant evidence for DEHP and related phthalates.

## 5 Conclusion

This study proposes a multi-scale, data-driven AOP linking environmental DEHP exposure to pancreatic ductal adenocarcinoma. Using a heterogeneous ensemble learning framework, we identified a consensus six-gene signature and prioritized KLF5 as a prognostically relevant and genetically supported candidate regulatory node. Molecular docking and 100-ns MD simulations support the *in silico* plausibility of a putative DEHP–KLF5 interaction within the modeled system, providing a candidate MIE that requires direct target-engagement validation. Single-cell and network-based perturbation analyses further indicate that KLF5 acts predominantly as a tumor-intrinsic regulator, consistent with an MMP7- and TGF-β-centered matrix-remodeling and immunomodulatory program.

*In vitro*, DEHP exposure upregulated KLF5 and MMP7 and enhanced pro-migratory and pro-invasive phenotypes in PANC-1 cells, providing functional support for a DEHP-associated KLF5/MMP7 branch as a cellular-level key event. Collectively, these findings support a working model in which DEHP-responsive KLF5 signaling aligns with metabolic/proliferative programs and extracellular matrix remodeling and is accompanied by a fibrotic, immune-attenuated stromal microenvironment inferred from deconvolution and single-cell communication analyses. Although key mechanistic links remain to be directly verified—particularly molecular target engagement, KLF5 dependency, and the predicted immune-cell consequences—this study illustrates how systems toxicology can connect environmental exposure to disease-relevant molecular networks and phenotypes, informing hazard identification for plasticizer-associated malignancies and motivating further interrogation of the KLF5 axis in environmentally driven PDAC progression.

## Data Availability

The original contributions presented in the study are included in the article/Supplementary Material; further inquiries can be directed to the corresponding author.

## Glossary

DEHP: Di(2-ethylhexyl) phthalate
MIEs: Molecular initiating events
AOP: Adverse outcome pathway
PDAC: Pancreatic ductal adenocarcinoma
PCV: Polyvinyl chloride
MEHP: Mono(2-ethylhexyl) phthalate
EDCs: Endocrine-disrupting chemicals
KLF5: Krüppel-like factor 5
TIME: Tumor immune microenvironment
MR: Mendelian randomization
MD: Molecular dynamics
SMILES: Simplified Molecular Input Line Entry System
OMIM: Online Mendelian Inheritance in Man
GO: Gene Ontology
KEGG: Kyoto Encyclopedia of Genes and Genomes
BP: Biological process
CC: Cellular component
MF: Molecular function
TabPFN: Tabular Prior-Data Fitted Network
SVM: Support vector machine
GBDT: Gradient boosting decision tree
XGBoost: eXtreme Gradient Boosting
SHAP: SHapley Additive exPlanations
TCGA: The Cancer Genome Atlas
GTEx: Genotype–tissue expression
OS: Overall survival
RMSD: Root mean square deviation
RMSF: Root mean square fluctuation
Rg: Radius of gyration
GRN: Gene regulatory network
Virtual KO: Virtual knockout
SD: Standard deviation
WoE: Weight of evidence
KE: Key event
KERs: Key event relationships
AO: Adverse outcome
qPCR: Quantitative real-time PCR.

## References

[B1] Ankley G. T. Bennett R. S. Erickson R. J. Hoff D. J. Hornung M. W. Johnson R. D. (2010). Adverse outcome pathways: a conceptual framework to support ecotoxicology research and risk assessment. Environ. Toxicol. Chem. 29 (3), 730–741. 10.1002/etc.34 20821501

[B2] Baralić K. Živančević K. Javorac D. Buha Djordjević A. Anđelković M. Jorgovanović D. (2020). Multi-strain probiotic ameliorated toxic effects of phthalates and bisphenol A mixture in wistar rats. Food Chem. Toxicol. 143, 111540. 10.1016/j.fct.2020.111540 32645469

[B3] Boehm F. J. Zhou X. (2022). Statistical methods for Mendelian randomization in genome-wide association studies: a review. Comput. Struct. Biotechnol. J. 20, 2338–2351. 10.1016/j.csbj.2022.05.015 35615025 PMC9123217

[B4] Chen F. P. Chien M. H. Lee C. H. (2023). Regulation of the cell cycle and P13K/AKT/mTOR signaling pathway by phthalates in normal human breast cells. Taiwan J. Obstet. Gynecol. 62 (3), 434–439. 10.1016/j.tjog.2022.08.020 37188449

[B5] Diaferia G. R. Balestrieri C. Prosperini E. Nicoli P. Spaggiari P. Zerbi A. (2016). Dissection of transcriptional and cis-regulatory control of differentiation in human pancreatic cancer. EMBO J. 35 (6), 595–617. 10.15252/embj.201592404 26769127 PMC4801945

[B6] Eberhardt J. Santos-Martins D. Tillack A. F. Forli S. (2021). AutoDock vina 1.2.0: new docking methods, expanded force field, and Python bindings. J. Chem. Inf. Model 61 (8), 3891–3898. 10.1021/acs.jcim.1c00203 34278794 PMC10683950

[B7] Elyada E. Bolisetty M. Laise P. Flynn W. F. Courtois E. T. Burkhart R. A. (2019). Cross-Species single-cell analysis of pancreatic ductal adenocarcinoma reveals antigen-presenting cancer-associated fibroblasts. Cancer Discov. 9 (8), 1102–1123. 10.1158/2159-8290.CD-19-0094 31197017 PMC6727976

[B8] Fey R. M. Nichols R. A. Tran T. T. Vandenbark A. A. Kulkarni R. P. (2024). MIF and CD74 as emerging biomarkers for immune checkpoint blockade therapy. Cancers (Basel) 16 (9), 1773. 10.3390/cancers16091773 38730725 PMC11082995

[B9] Ge P. Wang W. Li L. Zhang G. Gao Z. Tang Z. (2019). Profiles of immune cell infiltration and immune-related genes in the tumor microenvironment of colorectal cancer. Biomed. Pharmacother. 118, 109228. 10.1016/j.biopha.2019.109228 31351430

[B10] Gill D. Georgakis M. K. Koskeridis F. Jiang L. Feng Q. Wei W. Q. (2019). Use of genetic variants related to antihypertensive drugs to inform on efficacy and side effects. Circulation 140 (4), 270–279. 10.1161/CIRCULATIONAHA.118.038814 31234639 PMC6687408

[B11] Hao Y. Hao S. Andersen-Nissen E. Mauck W. M. 3rd Zheng S. Butler A. (2021). Integrated analysis of multimodal single-cell data. Cell 184 (13), 3573–3587.e29. 10.1016/j.cell.2021.04.048 34062119 PMC8238499

[B12] He P. Yang J. W. Yang V. W. Bialkowska A. B. (2018). Krüppel-like factor 5, increased in pancreatic ductal adenocarcinoma, promotes proliferation, acinar-to-ductal Metaplasia, pancreatic intraepithelial Neoplasia, and tumor growth in mice. Gastroenterology 154 (5), 1494–1508.e13. 10.1053/j.gastro.2017.12.005 29248441 PMC5880723

[B13] Hessmann E. Buchholz S. M. Demir I. E. Singh S. K. Gress T. M. Ellenrieder V. (2020). Microenvironmental determinants of pancreatic cancer. Physiol. Rev. 100 (4), 1707–1751. 10.1152/physrev.00042 32297835

[B14] Hollmann N. Müller S. Purucker L. Krishnakumar A. Körfer M. Hoo S. B. (2025). Accurate predictions on small data with a tabular foundation model. Nature 637 (8045), 319–326. 10.1038/s41586-024-08328-6 39780007 PMC11711098

[B15] Krstic J. Santibanez J. F. (2014). Transforming growth factor-beta and matrix metalloproteinases: functional interactions in tumor stroma-infiltrating myeloid cells. ScientificWorldJournal 2014, 521754. 10.1155/2014/521754 24578639 PMC3918721

[B16] Landkocz Y. Poupin P. Atienzar F. Vasseur P. (2011). Transcriptomic effects of di-(2-ethylhexyl)-phthalate in Syrian hamster embryo cells: an important role of early cytoskeleton disturbances in carcinogenesis? BMC Genomics 12, 524. 10.1186/1471-2164-12-524 22026506 PMC3218109

[B17] Lemkul J. A. (2024). Introductory tutorials for simulating protein dynamics with GROMACS. J. Phys. Chem. B 128 (39), 9418–9435. 10.1021/acs.jpcb.4c04901 39305267 PMC11457149

[B18] Li Y. Zhang B. Xiang L. Xia S. Kucuk O. Deng X. (2020). TGF-β causes Docetaxel resistance in Prostate Cancer *via* the induction of Bcl-2 by acetylated KLF5 and protein Stabilization. Theranostics 10 (17), 7656–7670. 10.7150/thno.44567 32685011 PMC7359077

[B19] Li R. Ji Q. Fu S. Gu J. Liu D. Wang L. (2025). ITGA3 promotes pancreatic cancer progression through HIF1α- and c-Myc-driven glycolysis in a collagen I-dependent autocrine manner. Cancer Gene Ther. 32 (2), 240–253. 10.1038/s41417-024-00864-7 39690180

[B20] Lin Y. Wei J. Li Y. Chen J. Zhou Z. Song L. (2011). Developmental exposure to di(2-ethylhexyl) phthalate impairs endocrine pancreas and leads to long-term adverse effects on glucose homeostasis in the rat. Am. J. Physiol. Endocrinol. Metab. 301 (3), E527–E538. 10.1152/ajpendo.00233.2011 21673306

[B21] Ma J. B. Bai J. Y. Zhang H. B. Jia J. Shi Q. Yang C. (2020). KLF5 inhibits STAT3 activity and tumor metastasis in prostate cancer by suppressing IGF1 transcription cooperatively with HDAC1. Cell Death Dis. 11 (6), 466. 10.1038/s41419-020-2671-1 32546700 PMC7297795

[B22] Mariana M. Cairrao E. (2023). The relationship between phthalates and diabetes: a review. Metabolites 13 (6), 746. 10.3390/metabo13060746 37367903 PMC10301313

[B23] Martínez-Pinna J. Sempere-Navarro R. Medina-Gali R. M. Fuentes E. Quesada I. Sargis R. M. (2023). Endocrine disruptors in plastics alter β-cell physiology and increase the risk of diabetes mellitus. Am. J. Physiol. Endocrinol. Metab. 324 (6), E488–E505. 10.1152/ajpendo.00068.2023 37134142 PMC10228669

[B24] Osorio D. Zhong Y. Li G. Xu Q. Yang Y. Tian Y. (2022). scTenifoldKnk: an efficient virtual knockout tool for gene function predictions *via* single-cell gene regulatory network perturbation. Patterns (N Y) 3 (3), 100434. 10.1016/j.patter.2022.100434 35510185 PMC9058914

[B25] Peduzzi G. Archibugi L. Farinella R. Ponz de Leon Pisani R. Vodickova L. Vodicka P. (2025). The exposome and pancreatic cancer, lifestyle and environmental risk factors for PDAC. Semin. Cancer Biol. 113, 100–129. 10.1016/j.semcancer.2025.05.004 40368260

[B26] Peng J. Sun B. F. Chen C. Y. Zhou J. Y. Chen Y. S. Chen H. (2019). Single-cell RNA-Seq highlights intra-tumoral heterogeneity and malignant progression in pancreatic ductal adenocarcinoma. Cell Res. 29 (9), 725–738. 10.1038/s41422-019-0195-y 31273297 PMC6796938

[B27] Qi X. Wang S. Fang C. Jia J. Lin L. Yuan T. (2025). Machine learning and SHAP value interpretation for predicting comorbidity of cardiovascular disease and cancer with dietary antioxidants. Redox Biol. 79, 103470. 10.1016/j.redox.2024.103470 39700695 PMC11729017

[B28] Sun X. Lin Y. Huang Q. Shi J. Qiu L. Kang M. (2015). Di(2-ethylhexyl) phthalate-induced apoptosis in rat INS-1 cells is dependent on activation of endoplasmic reticulum stress and suppression of antioxidant protection. J. Cell Mol. Med. 19 (3), 581–594. 10.1111/jcmm.12409 25418486 PMC4369815

[B29] Sung H. Ferlay J. Siegel R. L. Laversanne M. Soerjomataram I. Jemal A. (2021). Global cancer statistics 2020: GLOBOCAN estimates of incidence and mortality worldwide for 36 cancers in 185 countries. CA Cancer J. Clin. 71 (3), 209–249. 10.3322/caac.21660 33538338

[B30] Wang X. Qiu T. Wu Y. Yang C. Li Y. Du G. (2021). Arginine methyltransferase PRMT5 methylates and stabilizes KLF5 *via* decreasing its phosphorylation and ubiquitination to promote basal-like breast cancer. Cell Death Differ. 28 (10), 2931–2945. 10.1038/s41418-021-00793-0 33972717 PMC8481478

[B31] Wang A. Liu H. Yang J. Chen G. (2022). Ensemble feature selection for stable biomarker identification and cancer classification from microarray expression data. Comput. Biol. Med. 142, 105208. 10.1016/j.compbiomed.2021.105208 35016102

[B32] Wang S. Meng B. Liu Y. Wu Q. (2025). Identification of PDGFRB as a prognostic immune-related biomarker in gastric cancer through bioinformatics and clinical analysis. Transl. Cancer Res. 14 (10), 6849–6863. 10.21037/tcr-2025-859 41234850 PMC12605553

[B33] Wild C. P. (2012). The exposome: from concept to utility. Int. J. Epidemiol. 41 (1), 24–32. 10.1093/ije/dyr236 22296988

[B34] Zhang Y. Zhang Z. (2020). The history and advances in cancer immunotherapy: understanding the characteristics of tumor-infiltrating immune cells and their therapeutic implications. Cell Mol. Immunol. 17 (8), 807–821. 10.1038/s41423-020-0488-6 32612154 PMC7395159

[B35] Zhou Y. Tang Y. Huang F. Wang Z. Wen Z. Fang Q. (2025a). The miR-1305/KLF5 negative regulatory loop affects pancreatic cancer cell proliferation and apoptosis. Hum. Cell 38 (2), 51. 10.1007/s13577-025-01173-3 39921786

[B36] Zhou X. Ye W. Xu J. Luo Q. Huang Y. Li J. (2025b). The role of di-(2-ethylhexyl) phthalate in cancer initiation and progression: mechanisms and health implications. Sci. Total Environ. 959, 178285. 10.1016/j.scitotenv.2024.178285 39756301

